# Clinical characteristics and outcome of Covid-19 illness and predictors of in-hospital mortality in Saudi Arabia

**DOI:** 10.1186/s12879-022-07945-8

**Published:** 2022-12-17

**Authors:** Mostafa A. Abolfotouh, Abrar Musattat, Maha Alanazi, Suliman Alghnam, Mohammad Bosaeed

**Affiliations:** 1 King Abdullah International Medical Research Center, Mail Code 3533, Riyadh, Saudi Arabia; 2 King Saud Bin-Abdulaziz University for Health Sciences (KSAU-HS), Riyadh, Saudi Arabia; 3 King Abdulaziz Medical City, Ministry of National Guard-Health Affairs, POB 22490, Riyadh, 11426 Saudi Arabia

**Keywords:** SARS-CoV-2, Disease severity, ICU-admission, Hospitalization, Key parameters, Laboratory findings, Comorbidities, Case fatality, Risk factors, AUC

## Abstract

**Background:**

Patients’ race and ethnicity may play a role in mortality from Covid-19. Studies in China, the US, and Europe have been conducted on the predictors of Covid-19 mortality, yet in the EMR countries, such studies are scarce. Therefore, we aimed to describe the hospitalization rate, ICU-admission, and in-hospital mortality of Covid-19 and predictors of in-hospital mortality in Saudi Arabia.

**Methods:**

E-medical records were examined for all Covid-19 patients diagnosed in five tertiary hospitals affiliated with the Saudi-National Guard-Health Affairs during March 21, 2020, and September 12, 2021, based on a positive SARS-CoV-2 RT-PCR test, (n = 35,284). Data were collected on patients’ characteristics, comorbidities, laboratory findings, hospitalization, ICU admission, and in-hospital and overall mortality. Logestic regressions were used to identify the independent predictors of in-hospital mortality. The best laboratory parameters cut-off values to predict in-hospital mortality were identified using the area under the receiver operating characteristic curve (AUC). Significance was considered at p < 0.05.

**Results:**

Of all 35,284 Covid-19 patients, 81.8% were adults and 21.7% were hospitalized. Compared to non-hospitalized patients, hospitalized patients were more of female gender (52.1% versus 47.3%, p < 0.001) and had higher mean age (p < 0.001), higher mean BMI (p < 0.001), and higher rates of: diabetes (p < 0.001), hypertension (p < 0.001), ischemic heart disease (p < 0.001), cancer (p < 0.001), COPD (p < 0.001) and asthma (p = 0.011). The study showed 3.1% overall case-fatality, 20.3% ICU admission rate, and 9.7% in-hospital mortality. Predictors of in-hospital mortality among adult patients were; patients’ age ≥ 70 years (OR = 6.93, 95% CI 1.94–24.79), ischemic heart disease (OR = 1.80, 95% CI 1.05–3.09), ICU admission (OR = 24.38, 95% CI 15.64–38.01), abnormal C-reactive protein “CRP” (OR = 1.85, 95% CI 1.08–3.16), abnormal D-dimer (OR = 1.96, 95% CI 1.15–3.36), lymphopenia (OR = 2.76, 95% CI 2.03–3.3.76), high neutrophil count (OR = 2.10, 95% CI 1.54–2.87), and abnormal procalcitonin (OR = 3.33, 95% CI 1.88–5.90). The best laboratory parameters cut-off values to predict in-hospital mortality were CRP > 72.25 mg/L (AUC = 0.64), d-dimer > 1125 µg/L (AUC = 0.75), neutrophils count > 5,745 × 10^9/L (AUC = 0.70), lymphocytic count < 1.10 × 10^9/L (AUC = 0.72), and procalcitonin > 0.18 ng/mL (AUC = 0.76).

**Conclusions:**

Rates of hospitalization, ICU-admission, in-hospital mortality and overall case fatality were nearly comparable to the rates in western countries. Early interventions are necessary for high-risk Covid-19 patients, especially elderly patients and those with cardiac diseases.

## Background

Coronavirus disease 2019 (COVID-19) is a respiratory transmissible disease caused by the virus of SARS-CoV-2. The spread of the virus began in late 2019, and as of January 2, 2022, over two hundred and eighty million people are infected, relating to more than five million deaths worldwide [1]. Many researchers have studied factors about the mortality of patients diagnosed with COVID-19. One of the most frequent predictors was age; multiple studies concluded that increased age was associated with higher mortality [2–4]. A meta-analysis of 49 articles and over 20,000 patients concluded that age 50 and above had a strong association with these patients’ mortality [5]. Moreover, comorbidities predicted higher mortality in moderate to severe COVID-19 patients [6]; included hypertension, diabetes mellitus, ischemic heart disease, cancer, chronic kidney disease, and liver injuries.

Specific laboratory parameters were associated with an increased risk of mortality. For instance, elevated C-reactive protein (CRP) and d-dimer levels were associated with increased mortality odds [6–9]. According to Leonidas Palaiodimos et al. [10], severe obesity with a high body mass index of more than 35 kg/m^2^ was associated with higher in-hospital mortality among COVID-19 patients.

Patients’ race and ethnicity may play a role in mortality from Covid-19. Mortality rates among Black Americans were 92.3 deaths/100,000 population and 74.3 deaths/100,000 among Hispanic Americans, higher than those of white Americans (45.2 deaths/100,000) or Asians (34.5 deaths/100,000) [11]. Economic and social conditions also may play a role [12, 13]. Living in heavily crowded areas may be a barrier to prevention measures like social distancing. In addition, economic status in different countries may influence the outcomes due to difference in resources needed for management [14–16]. Although a fair number of articles related to Covid-19 mortality predictors in Saudi Arabia were published [7, 9, 13, 17, 18], none of these studies was found with a large sample size that guarantees its validity. This study aimed to describe, in a large cohort of Saudi patients, the characteristics and outcomes of Covid-19 illness in children and adults, and to determine the predictors of in-hospital mortality in adult patients.

## Methods

### Study Design and study subjects

This is a retrospective cohort study of all patients diagnosed and treated in five tertiary hospitals affiliated with the Saudi Ministry of National Guard-health affairs (MNG-HA), from the start of the virus spread in Saudi Arabia on March 21, 2020, until September 12, 2021 (n = 35,284). All individuals with suspected Covid-19 symptoms, and those with history of a recent exposure to a Covid-19 case, in those five hospitals, were screened for Covid-19. Diagnosis was made based on a positive SARS-CoV-2 Reverse transcription-polymerase chain reaction (RT-PCR) from nasopharyngeal swabs.

### Study setting

MNG-HA provides healthcare services to national guard service members and their dependents through large medical cities located in the three most densely populated regions of Saudi Arabia, namely the Central (one hospital), Western (two hospitals), and Eastern (two hospitals) regions. All facilities have been Joint Commission International (JCI)-accredited since 2006. During the COVID-19, and following the first reported case in Saudi Arabia, MNG-HA has taken drastic infection control measures that included the reduction of elective surgeries, stopping in person outpatient services, and introducing ER workflow to minimize Covid19 cases flow through the main ER [19]. Standard treatment of patients infected with COVID-19 includes symptoms treatment, supportive measures such as; supplemental oxygen, and approved drugs to treat the infection per the MOH and MNGHA treatment guidelines [20]. Recommendations for adult patients admitted to the ICU include antiviral therapy, Immunomodulation therapy, and corticosteroids. Dexamethasone over other corticosteroids is also suggested [21].

### Data collection

The collected variables included routine demographic variables such as age and sex, and location, Comorbidities included Diabetes, Hypertension, Cancer, Chronic obstructive pulmonary disease, Asthma, and Ischemic heart disease, biometric measurements, i.e., Body-Mass-Index, Laboratory data including C-reactive protein (CRP), D-dimer, Ferritin, Lactate Dehydrogenase (LDH), Lymphocytes, Neutrophils, and Procalcitonin, and outcome variables including hospitalization, intensive care unit (ICU) admission, mortality status, in-hospital mortality, and time from diagnosis to death.

Data was extracted from the health system’s electronic Datawarehouse, which host all medical information for patient care. Based on the study inclusion criteria, the data was extracted using a search engine within the Electronic Health Record (EHR) and collated in a structured format including ICD codes, lab values and health utilization (i.e., ICU admission). All the data obtained are documented by the healthcare professionals who provided those services to patients. All hospitals, within the MNG-HA, provide uniform healthcare services across the organization. Therefore, data collected are consistent across hospitals. Additional validation for mortality status was done by ensuring other information are documented, such as date and time of death, and the presence of a death notice.

### Ethical issues

The study was approved by the Institutional Review Board (IRB) of the Ministry of National Guard- Health Affairs (MNG-HA) by study number NRC21R/445/10. The IRB of the MNG-HA waived the requirement for informed consent because of the retrospective nature of the study. All methods were carried out in accordance with relevant guidelines and regulations.

### Data analysis

Data were analyzed using the (SPSS version 26.0; IBM Corporation, Armonk, NY, USA). We used descriptive statistics such as mean (SD), median (IQR), and range. Frequencies (%) and their corresponding 95% confidence intervals (CIs) were estimated. Student *t* test was used to compare numerical data, while the chi-square test and Fisher exact test were applied for categorical data. Odds ratios (ORs) and their corresponding 95% CIs were calculated, and logistic regression analysis was applied to adjust for confounders of the association between different variables and the in-hospital case fatality of Covid-19. Kolmogorov–Smirnov and Shapiro–Wilk tests were applied to test for normality of age and BMI variables before deciding which statistical test to use. Receiver Operator Characteristic curve (ROC) was applied to allocate the optimum cut-off values for age and different laboratory parameters in the prediction of in-hospital mortality from Covid-19. For every parameter optimum cut-off, accuracy was determined, in terms of sensitivity and specificity, with the corresponding 95% confidence intervals, and the area under the curve (AUC). Significance was considered at p ≤ 0.05.

The primary outcomes were the characteristics and outcome of Covid-19 illness. The secondary outcome was predictors of in-hospital mortality to help for proper and timely treatment of patients with Covid-19 illness.

## Results

### Hospitalization & In-hospital mortality rate among Covid-19 patients

Of all 35,284 Covid-19 patients, 28,876 patients (81.8%) were adults 18 years or more and 6408 patients (18.2%) were children less than 18 years. A total of 7484 patients (21.7%) were hospitalized [24.3% of adult patients and 9.7% of children with Covid-19, χ^2^ = 664.15, p < 0.001]. Of all hospitalized patients, 1552 patients (20.3%) were admitted to ICU [21.1% of hospitalized adult patients and 11.5% of hospitalized children, χ^2^ = 32.61, p < 0.001]. The overall case fatality rate was 3.1% [3.7% of adults and 0.3% of children, χ^2^ = 206.97, p < 0.001]. In-hospital mortality rate was 9.7% of all hospitalized patients [10.5% of hospitalized adults and 1.1% of hospitalized children, χ^2^ = 57.00, p < 0.001], Table 1.

**Table 1:** Distribution of all Covid-19 patients (n = 35,284) diagnosed in the Ministry of National Guard Hospitals, Saudi Arabia [March 21, 2020 until September 12, 2021] according to age, hospitalization, ICU admission and in-hospital mortality

Characteristics	Total No (%)	Child (< 18 yrs) No (%)	Adult [≥ 18 yrs] No(%)	p-value^@^
	35,284 (100.0)	6408 (18.2)	28,876 (81.8)	
Male gender	18,226 (51.7)	3352 (52.3)	14,874 (51.5	0.25
Hospitalization	7484 (21.7)	620 (9.7)	7028 (24.3)	< 0.001
ICU admission^#^	1552 (20.3)	71 (11.5)	1481 (21.1)	< 0.001
LOS in days [Median (IQR)]		5(2–14)	9 (4–18)	
In-hospital mortality^#^	746 (9.7)	7 (1.1)	739 (10.5)	< 0.001
LOS in days [Median (IQR)]		16.0 (4–37)	16 (7–27)	
Overall case fatality	1093 (3.1)	18 (0.3)	1075 (3.7)	< 0.001

^@^Pearson Chi square test was applied; ^#^Percentage was estimated out of the total number of hospitalizations. LOS: length of stay; IQR: interquartile range

### Personal characteristics and comorbidities among adult Covid-19 patients

Of all hospitalized patients, 51.5% were males and 48.5% were females. Most patients (43.0%) were 30 to < 50 years of age, and 30.9% of patients were ≥ 50 years of age, with a mean age of 42.4 ± 16.7 years. The majority of patients had a BMI of ≥ 30 kg/m^2^. The most common comorbidities were diabetes (14.4%) and hypertension (11.8%). Hospitalized patients were significantly older (54.4 ± 4 years versus 38.5 ± 14.1 years, p < 0.001) and with higher BMI (p < 0.001). Concerning comorbidities, hospitalized patients showed a significantly higher rate of diabetes (p < 0.001), hypertension (p < 0.001), ischemic heat disease (p < 0.001), cancer (p < 0.001), COPD (p < 0.001), and Asthma (p = 0.011). There was a significant sex difference between hospitalized and non-hospitalized patients, with higher proportion of females among hospitalized patients (p < 0.001), Table 2.

**Table 2:** Distribution of hospitalized and non-hospitalized adult Covid-19 patients according to personal characteristics and comorbidity

	Total No (%)	Unhospitalized No (%)	Hospitalized No (%)	P-value
ALL	28,876 (100.0)	21,848 (75.7)	7028 (24.3)	
Male gender	14,874 (51.5)	11,508 (52.7)	3366 (47.9)	< 0.001^@^
Age group (years)				
< 30	7529 (26.1)	6724 (30.8)	805 (11.5)	
30–	12,424 (43.0)	10,497 (48.0)	1927 (27.4)	
50–	6597 (22.8)	3915 (17.9)	2682 (38.2)	
70 or more	2326 (8.1)	712 (3.3)	1614 (23.0)	< 0.001^@^
Mean (SD)	42.4 ± 16.7	38.5 ± 14.1	54.4 ± 18.4	< 0.001^#^
BMI (kg/m^2^)				
< 25	3244 (22.3)	2300 (23.5)	944(19.8)	
25–	4562 (31.3)	3169(32.4)	1393 (29.2)	
30 or more	6762 (46.4)	4323(44.1)	2439 (51.1)	< 0.001^@^
Mean (SD)	29.9 ± 6.3	29.6 ± 6.2	30.6 ± 6.5	< 0.001^#^
Comorbidities				
Diabetes	4161 (14.4)	2191 (10.0)	1970 (28.0)	< 0.001^@^
Hypertension	3412 (11.8)	1769 (8.1)	1643 (23.4)	< 0.001^@^
Asthma	701 (2.4)	502 (2.3)	199 (2.8)	0.011^@^
COPD	63 (0.2)	8 (0.0)	55 (0.8)	< 0.001^@^
Ischemic heart disease	637 (2.2)	242 (1.1)	395 (5.6)	< 0.001^@^
Cancer	739 (2.6)	298 (1.4)	441 (6.3)	< 0.001^@^

^@^Pearson chi square test was applied; ^#^student *t*-test was applied

### Predictors of in-hospital mortality

In bivariate analysis, the rate of in-hospital mortality was significantly higher among male patients (p < 0.001), patients ages 30 to < 50 years (p = 0.006), 50 to < 70 years (p < 0.001) and ≥ 70 years of age (p < 0.001). Obese patients of ≥ 30 kg/m^2^ showed significantly lower in-hospital mortality (p = 0.001). However, after adjusting for confounders, old age of 70 or more was the only significant personal factor associated with in-hospital mortality among hospitalized Covid-19 patients (OR = 6.93, 95% CI 1.94–24.79, p = 0.003), Table 3.

**Table 3:** In-hospital mortality rate (%) in adult hospitalized Covid-19 patients according to some personal characteristics, comorbidities and laboratory findings

	Inhospital mortality No (%)	cOR (95% CI)	P value	aOR (95% CI)	P-value
A. Personal characteristics					
Gender					
Female	276 (7.5)	1		1	
Male	462 (13.7)	1.95 (1.67–2.28)	< 0.001*	1.05 (0.75–1.48)	0.76
Age (years)					
< 30	10 (1.2)	1		1	
30–	59 (3.1)	2.51 (1.28–4.93)*	0.006*	1.40 (0.37–5.23)	0.62
50–	306 (11.4)	10.24 (5.43–19.32)*	< 0.001*	3.15 (0.89–11.14)	0.08
70 or more	363 (22.5)	23.07 (12.23–43.51)*	< 0.001*	6.93 (1.94–24.79)	0.003*
BMI (kg/m^2^)					
< 25	166 (17.6)	1		1	
25–	213 (15.3)	0.85 (0.68–1.06)	0.14	1.22 (0.78–1.90)	0.40
30 or more	302 (12.4)	0.66 (0.54–0.82)*	0.001*	1.25 (0.81–1.94)	0.31
B. Comorbidities					
Diabetes					
No	493 (9.7)	1		1	
Yes	245 (12.4)	1.32 (1.12–1.55)*	0.001	1.04 (0.71–1.52)	0.85
Hypertension					
No	510 (9.5)	1		1	
Yes	228 (13.9	1.54 (1.30–1.82)*	< 0.001	0.78 (0.52–1.16)	0.22
Ischemic heart disease					
No	669 (10.1)	1		1	
Yes	69 (17.5)	1.89 (1.44–2.48)*	< 0.001	1.80 (1.05–3.09)*	0.034*
Cancer					
No	677 (10.3)	1		1	
Yes	61 (13.8)	1.40 (1.06–1.86)*	0.018*	1.73 (0.99–3.02)	0.053
COPD					
No	726 (10.4)	1		1	
Yes	12 (21.8)	2.40 (1.26–4.58)*	0.006*	0.60 (0.19–1.90)	0.38
Asthma					
No	720 (10.5)	1		1	
Yes	18 (9.0)	0.84 (0.52–1.38)	0.50	1.41(0.60–3.37)	0.44
ICU adm					
No	165 (3.0)	1		1	
Yes	573 (38.7)	20.58 (17.08–24.81)*	< 0.001*	24.38 (15.64–38.01)*	< 0.001*
C. Laboratory findings					
CRP					
Normal	43 (4.6)	1		1	
Abnormal	549 (14.9)	3.62 (2.63–4.98)*	< 0.001*	1.85 (1.08–3.16)*	0.024*
d-Dimer					
Normal	36 (2.7)	1		1	
Abnormal	583 (16.2)	7.06 (5.01–9.95)*	< 0.001*	1.96 (1.15–3.36)*	0.014*
Ferritin					
Normal	87 (5.1)	1		1	
Abnormal	564 (15.9)	3.50 (2.77–4.42)*	< 0.001*	1.40 (0.91–2.16)	0.13
LDH					
Normal	13 (1.7)	1		1	
Abnormal	656 (1`4.7)	9.97 (5.73–17.36)*	< 0.001*	2.56 (0.82–7.98)	0.10
Lymphocytes					
Normal	267 (6.3)	1		1	
Decreased	400 (21.5)	4.06 (3.44–4.79)*	< 0.001*	2.76 (2.03–3.3.76)*	< 0.001*
Increased	4 (7.5)	1.21 (0.43–3.38)		0.000	0.99
Neutrophils					
Normal	279 (7.0)	1		1	
Increased	368 (25.3)	4.49 (3.79–5.32)*	< 0.001*	2.10 (1.54–2.87)*	< 0.001*
Decreased	28 (4.0)	0.55 (0.37–0.82)		1.04 (0.45–2.41)	0.93
Procalcitinon					
Normal	32 (3.5)	1		1	
Abnormal	564 (18.7)	6.30 (4.38–9.08)*	< 0.001*	3.33 (1.88–5.90)*	< 0.001*

cOR: crude odds ratio; aOR: adjusted odds ratio; *Statistically significant, LDH: Lactate Dehydrogenase, CRP: c-reactive protein; COPD: chronic obstructive pulmonary disease

In-hospital mortality rate was significantly higher among patients with diabetes (p = 0.001), hypertension (p < 0.001), IHD (p < 0.001), cancer (p = 0.018), and COPD (p = 0.006). After adjusting for possible confounders, of all comorbidities studied, IHD was only combidity significantly associated with in-hospital mortality (OR = 1.80, 95% CI 1.05–3.09, p = 0.034). Patients admitted to ICU showed significant association with in-hospital mortality (p < 0.001). Even after controlling for possible confounders, ICU admission remained a significant predictor of in-hospital mortality (OR = 24.38, 95% CI 15.64–38.01, p < 0.001), Table 3.

As for laboratory findings, after adjusting for possible confounders, in-hospital mortality was significantly associated with abnormal values of CRP (OR = 1.85, 95% CI 1.08–3.16, p = 0.024), d-dimer (OR = 1.96, 95% CI 1.15–3.36, p = 0.014), procalcitonin (OR = 3.33, 95% CI 1.88–5.90, p < 0.001), low lymphocyte count (OR = 2.76, 95% CI 2.03–3.3.76, p < 0.001), and high neutrophil count (OR = 2.10, 95% CI 1.54–2.87, p < 0.001), Table 3.

The ROC curves for age, CRP, D-dimer, procalcitonin, lymphocyte count and neutrophil count were applied to better predict in-hospital mortality. The best cut-off point for age was 64 years with a sensitivity of 64% and specificity of 60%, and AUC = 69%. The best laboratory findings cut-off points for prediction of in-hospital mortality were 72.25 mg/L for CRP, > 1125 µg/L for d-dimer, > 5,745 for neutrophils count, < 1.10 for the lymphocytic count and > 0.18 for procalcitonin, Table 4.

**Table 4:** Accuracy of predictors of in-hospital mortality in Covid-19 adult hospitalized patients as determined by the receiver operating characteristic curve (ROC)

Predictors	Cut-off value	Sensitivity (95% CI)	Specificity (95% CI)	AUC
Age (years)	64	0.68 [0.65–0.71]	0.71 [0.69–0.72]	0.69
CRP (mg/L)	72.25	0.62 [0.58–0.65]	0.66 [0.65–0.68]	0.64
d-dimer (µg/L)	1125	0.73 [0.70–0.76]	0.66 [0.64–0.67]	0.75
Lymphocytes (× 10^9/L)	1.10	0.65 [0.61–0.68]	0.68 [0.67–0.70]	0.72
Neutrophils (× 10^9/L)	5,745	0.66 [0.63–0.69]	0.67 [0.65–0.68]	0.70
Procalcitinon (ng/mL)	0.18	0.71 [0.68–0.74]	0.70 [0.68–0.72]	0.76

AUC: area under the curve; CI: confidence interval

## Discussion

Worldwide, a case fatality rate of 3.5% was estimated for Covid-19 [22], and this rate was similar to that of Spanish influenza (2–3%) but much higher than that of seasonal influenza (0.1%) [23]. Variable mortality rates for Covid-19, ranging from 18.9% to 0.1% [24], have been reported in different countries. The overall case fatality rate was high in Belgium [25], in US [26], and in UK [27]. Our study showed an overall case fatality rate of 3.1%, a figure that is comparable to figures in EMR countries (3.8%, 95% CI 3.8–3.9%) [24], yet much higher than the figures reported for the GCC countries (0.6%, 95% CI 0.55–0.65%) [28]. It was also comparable to rates in China [29] and Italy [30]. Worldwide, over 80% of COVID-19 cases have mild symptoms; however 10 and 20% of COVID-19 cases proceed to a severe stage [31]. This pattern was similar to the situation shown in our study, where 24.3% of all patients with Covid-19 were hospitalized because of moderate to severe symptoms, while 75.7% of cases did not need hospitalization, probably because of their mild symptoms.

Reported mortality rates of COVID-19 patients are in the range of 20–40% for hospitalised patients and 30–88% for critically-ill or ICU patients with substantial differences between countries and regions [32]. This diversity was attributed to the different strategies of referral to hospital, admission strategies and decisions on treatment withdrawal [32]. In-hospital mortality rate among hospitalized cases, in our study, was 9.7%. The incidence of fatalities in many developing countries appeared to be low in the early stages of the pandemic, due to their relatively younger age structure, poor vital statistics systems leading to underreporting of COVID-19 deaths, and lower access to good-quality healthcare [33]. However, this figure, in our study, is high when compared with the counterpart figure of 1.63% in Abu Dhabi, UAE [34].

ICU utilization may be viewed as an indicator of the quality of healthcare delivered. The global rates of 20–30% of Covid-19 ICU admissions were reported [35]. The overall rate of ICU admission in KSA in all regions for all COVID-19 patients who were admitted to hospital based on MOH criteria is almost was 50% [36]. In our study, this rate of ICU admission was 20.3% of all hospitalized patients. It was high compared to a rate of only 7.68% patients in Abu Dhabi. ICU admission rate is a sensitive indicator for the severity of the illness. In our study, the rate of in-hospital mortality among ICU admitted cases was 38.7%, a rate which is nearly two times the rate of 19.56% in Abu Dhabi. This difference might be attributed to the difference in the strategy of hospitalization in the two countries, where in Abu Dhabi, initially, all people with a positive PCR were admitted regardless of clinical presentation for isolation and monitoring, a strategy that Saudi Arabia didn’t practice.

Patients’ characteristics, comorbidities and laboratory values have been reported and considered in the recent guidelines for the management of Covid-19 [35, 37–39]. Age was reported as the most significant predictor of mortality in patients with Covid-19 [40–50]. In our study, the old age of 70 or more was the only significant personal factor associated with in-hospital mortality among hospitalized Covid-19 patients. Older patients showed higher tendency to progress to severe Covid-1 9 illness than relatively younger patients [21], and this was attributed to functional defects in immune cells, leading to inability to suppress viral replication, as age advances [45–47]. In our study, age of > 64 was the significant cut-off age for the prediction of in-hospital mortality from Covid-19. This finding was in agreement with the finding of a cut-off value of ≥ 65 years reported in a previous study [51].

Some studies reported that obesity was a significant predictor of mortality from Covid-19 [52–54]. However, in our research, adjusting for other possible confounders, obesity was not amongst the predictors of mortality. This finding was in agreement with the results of Stefan et.al [25]. The association of obesity with lower mortality could be attributed to the possible natural protective effect of adipose tissue [55, 56], or to the lower levels of proinflammatory cytokines in obese subjects.

Hypertension, diabetes, and coronary heart disease were the most common comorbidities among Covid-19 patients, in our study. These findings are similar to the common comorbidities observed in the US, Italy, China, and UAE [34, 57, 58]. The following comorbidities were reported as key predictors for Covid-cases progression: Diabetes mellitus [59–61], hypertension [60–63], COPD [59], and Ischemic heart disease [43]. In our study, of all these comorbidities, ischaemic heart disease was the only significant predictor of in-hospital mortality, and Covid-19 cases who suffered from ischemic heart disease were two times more likely to end with in-hospital mortality. In a nested case–control study to evaluate the risk of pre-existing comorbidities on Covid-19 mortality [64], in-hospital mortality was three times more likely to occur among cases with pre-existing cardiac disease than those without. Early medical intervention is necessary for Covid-19 patients with pre-existing comorbidities, especially cardiac disease.

In terms of laboratory results, the following abnormal laboratory findings were reported as key predictors for Covid-19 cases progression; d-dimer [59, 60, 65–68], CRP [59, 64, 67–71], LDH [60, 72–76], lymphocytes [59, 60, 62, 68–70, 73, 74, 77], and procalcitonin levels [78]. In our study, in-hospital mortality was significantly associated with abnormal values of CRP, with a cut-off of 72.25 mg/L (AUC = 0.64). CRP has been reported as a significant predictor of severe Covid-19 illness [79], and an early marker of infection and inflammation [80]. In our study, d-dimer was a significant predictor of in-hospital mortality, with a cut-off value of > 1125 µg/L (AUC = 0.75). Increased d-dimer levels was associated with microthrombi formation, resulting from viral management of vascular endothelial cells [81]. Increased procalcitonin levels was associated with severe bacterial infection, and progression to severe condition in Covid-19 patients [78]. In our study, abnormal procalcitonin level was a predictor of, with a cut-off value of > 0.18 ng/mL (AUC = 0.76).

The pathogenesis of highly pathogenic human coronavirus is still not completely understood. Cytokine storm and viral evasion of cellular immune responses are thought to play important roles in disease severity [82]. Low lymphotic count was a significant predictor of in-hospital mortality, with a cut-off value of < 1.10 × 10^9/L (AUC = 0.72). Lymphocytes were reported to help clear SARS-CoV, and a suboptimal T-cell response was found to cause pathological changes observed in mice with SARS-CoV [83]. High neutrophil count was a significant predictor of in-hospital mortality as well, with a cut-off value of > 5,745 × 10^9/L (AUC = 0.70). Neutrophils are the main source of chemokines and cytokines. The generation of cytokine storm can lead to ARDS, which is a leading cause of death in patients with severe acute respiratory syndrome^r^ and Middle East respiratory syndrome [84]. However, further studies are needed to characterize the role of the neutrophil and lymphocyte response in SARS-CoV-2 infection. These indicators would help in early prediction and guide for proper, timely treatment.

## Limitations

This study has some limitations. First, it is a retrospective one, and the causal relationship between abnormal clinical/ laboratory indicators and death is not guaranteed. Second, the data were collected from hospitals affiliated with one institution (MNG-HA) in Saudi Arabia. However, these hospitals are five large tertiary, JCI-accredited hospitals across the whole Saudi Arabia. Moreover, the strategy of managing Covid-19 patients is the same in these tertiary hospitals, which would avoid the competing mortality risk caused by insufficient healthcare resources. Third, because not all patient profiles were complete, however, with a large cohort of more than 35 thousand patients in this study, we assumed that missing data are missing at random. Lastly, different clinical features or mechanisms for the disease progression might be promoted with new mutants SARS-coV-2. Thus, further prospective studies are needed to validate the present study’s findings.

## Conclusion

This study is one of the first observational studies in the EMR to determine the outcomes of Covid-19 disease and identify the key predictors of in-hospital mortality, with a large cohort of Covid-19 patients. The rates of hospitalization, ICU admission, in-hospital and overall mortality are nearly comparable to the counterpart rates in western countries. Older patients are more likely to die from Covid-19 illness than those of younger age. A pre-existing history of ischemic heart disease was the only comorbidity that predicts in-hospital mortality from Covid-19. Early interventions are required for Covid-19 patients with comorbidities, especially cardiac diseases. Careful monitoring of laboratory parameters in Covid-19 illness is necessary, with special attention to CRP, D-dimer, procalcitonin values, and lymphocyte and neutrophil counts.

## Data Availability

Most of the data supporting our findings is contained within the manuscript, and all others, excluding identifying/confidential patient data, will be shared upon request, by contacting the corresponding author [Mostafa Abolfotouh, mabolfotouh@gmail.com].

## Abbreviations

RT-PCR: _Reverse transcription-polymerase chain reaction
JCI: Joint Commission International
ER: Emergency department
MOH: Ministry of Health
ICUL: Intensive care unit
CRP: C-reactive protein
LDH: Lactate Dehydrogenase
IQR: Interquartile range
CI: Confidence interval
OR: Odds ratio
COPD: Chronic obstructive pulmonary disease
ROC: Receiver operating characteristic curve
AUC: Area under the curve
EMR: Eastern Mediterranean Region
GCC: Gulf Cooperation Council
UAE: United Arab Emarates
KAMC: King Abdulaziz Medical city
MNG-HA: Ministry of National Guard-Health Affairs
Covid-19: Coronavirus disease 2019
SARS-CoV-2: Severe Acute Respiratory Syndrome Coronavirus-2
WHO: World Health Organization
IHD: Ischemic heart disease
CFR: Case fatality rate
ER: Emergency department
ICU: Intensive care unit
IRB: Institutional Review Board
